# Gain-Scheduled Complementary Filter Design for a MEMS Based Attitude and Heading Reference System

**DOI:** 10.3390/s110403816

**Published:** 2011-03-29

**Authors:** Tae Suk Yoo, Sung Kyung Hong, Hyok Min Yoon, Sungsu Park

**Affiliations:** 1 Department of Aerospace Engineering, Sejong University, Seoul 143-747, Korea; E-Mail: 62187390@hanmail.net (T.S.Y.); 2 Youngpoong Electronics Co., Changwon, Kyungnam 641-847, Korea; E-Mail: hmyoon@ypelec.co.kr

**Keywords:** AHRS, complementary filter, gain-scheduling, inertial sensor, magnetic sensor

## Abstract

This paper describes a robust and simple algorithm for an attitude and heading reference system (AHRS) based on low-cost MEMS inertial and magnetic sensors. The proposed approach relies on a gain-scheduled complementary filter, augmented by an acceleration-based switching architecture to yield robust performance, even when the vehicle is subject to strong accelerations. Experimental results are provided for a road captive test during which the vehicle dynamics are in high-acceleration mode and the performance of the proposed filter is evaluated against the output from a conventional linear complementary filter.

## 1. Introduction

Aircraft, especially autonomous Unmanned Aerial Vehicles (UAVs), need to know their pose estimation for the lowest control and stabilization loops. When cost or weight is an issue, a low-cost Attitude and Heading Reference System (AHRS)—relying on MEMS inertial (gyroscopes and accelerometers) and magnetic sensors—plays an important role providing the vehicle’s orientation information relative to the inertial frame. In this respect, it has been of interest to develop robust and simple algorithms for reliable MEMS AHRS for the small scale embedded processors used in UAV avionic systems [1–9].

The two methods that are commonly used are Extended Kalman Filtering (EKF) or some form of constant gain state observer, often termed a complementary filter due to its frequency filtering properties for linear systems. Extended Kalman Filtering has been studied for a range of aerospace applications. Such filters, however, are computationally demanding and difficult to apply robustly [1–3]. In practice, many applications use simple linear single-input single-output (SISO) complementary filters. In recent work, a number of authors have developed nonlinear analogs of SISO filters for attitude estimation [4–9]. They approximate the accelerometer measurements via the measurement of gravity, so that a ‘weak acceleration’ assumption is made. Such fusion may allow obtaining a more accurate and less noisy attitude and heading estimation thanks to the sensor’s complementary characteristics. The filter fails, however, when vehicle dynamics are sufficiently high enough that the accelerometer output no longer provides a good estimate of the gravitational direction. This is particularly so when a UAV is circling for an extended period since the accelerometer, in this case, will detect not only gravitational acceleration but also centrifugal forces, resulting in an incorrect attitude determination [10].

In this paper, to cope with these situations, a gain-scheduled complementary filter, augmented by an acceleration-based switching architecture is proposed that yields robust performance, even when the vehicle is subject to strong acceleration. The gains of the proposed scheme are automatically tuned based on the system dynamic mode (non-acceleration, low-acceleration, and high acceleration mode) sensed by the accelerometers. Hence the system produces robust estimates of vehicle attitude and heading and removes the gyros bias that is a common source of drift error for both dynamic and stationary modes [11,12]. The ADIS16405 MEMS IMU (Inertial Measurement Unit) with magnetic sensors [13] from Analog Devices Inc. was selected for this research. The AHRS with a resulting filter structure is implemented in a real-time DSP (Digital Signal Processor) within a 5 cm × 4 cm × 3 cm size unit. Measurements of inertial sensors’ output obtained during the experimental road captive test are used for implementation of the estimation scheme. The estimation results were compared with the measurements from the on-board high-precision vertical gyro as it was used as the reference standard or ‘truth model’ for this analysis. This comparison explicitly demonstrates that more accurate AHRS performance can be achieved through the proposed switching architecture based integrated filtering estimator, even though low-cost MEMS inertial and magnetic sensors are used.

## 2. Attitude and Heading Determination from Inertial and Magnetic Sensors

### 2.1. Experimental Road Captive Test Data

**Figure 1.**
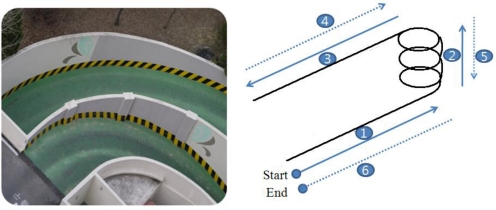
Test Scenario.

During the entire scheme investigation process in this paper, a set of road captive test data is used. As shown in Figure 1, the test runs were performed in a spiral structured parking building and the test scenario comprised the following procedures: (1) start (to induce linear acceleration), (2) spiral up (centrifugal acceleration), (3) forward (linear acceleration), (4) backward (linear acceleration), (5) spiral down (centrifugal acceleration), (6) forward (linear acceleration), and stop.

**Figure 2.**
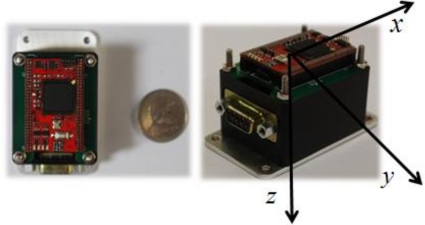
ADIS16405 DSP Module.

The commercial off-the-shelf IMU ADIS16405 used in the experiment is composed of three accelerometers, three gyros and three magnetometers with digital interface outputs. This inertial unit provides measurements of linear accelerations, angular rates, and the Earth’s magnetic fields for each body axis. Table 1 summarizes the basic specifications of each sensor. An AHRS prototype (Figure 2) was made using a DSP processor for the proposed estimator algorithm. The data acquisition was performed through a RS-232 communication serial port.

**Figure 3.**
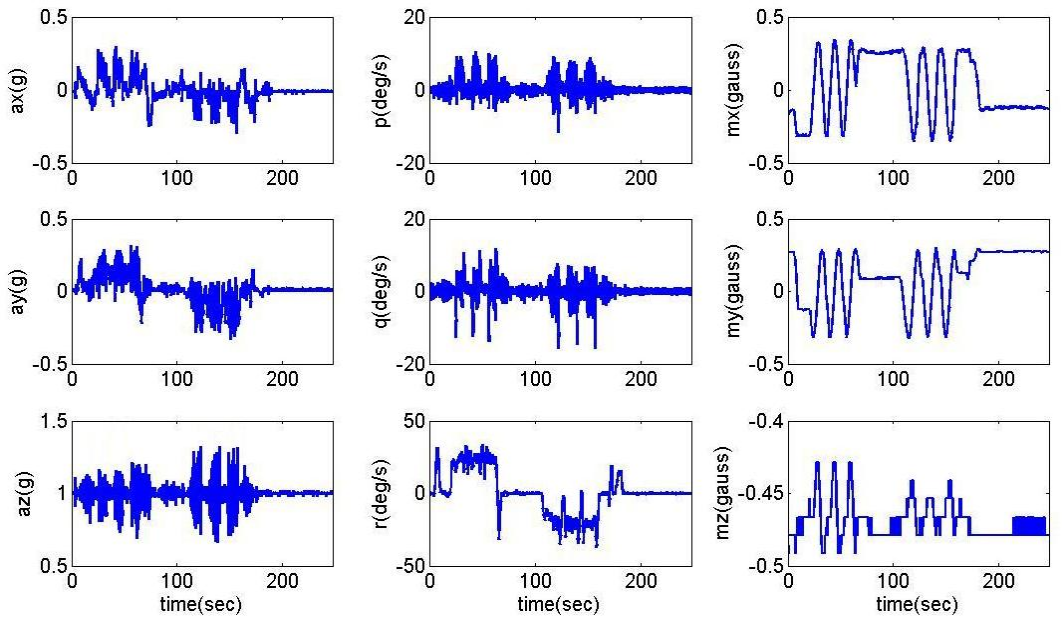
Raw data from ADIS16405.

**Figure 4.**
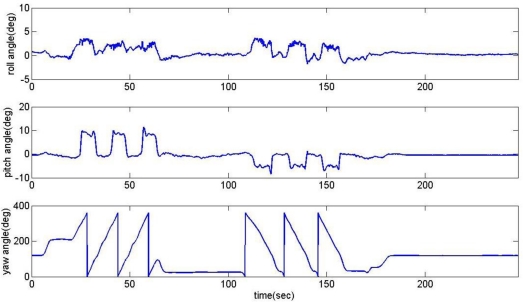
Attitude and heading angles from the on-board vertical gyro (reference standard).

The measurements of accelerations (*a_x_*, *a_y_*, *a_z_*), angular rates (*p*, *q*, *r*), and magnetic fields (*m_x_*, *m_y_*, *m_z_*) during the road captive test are shown in Figure 3. The reference attitude and heading angles for this analysis obtained from the on-board vertical gyro are shown in Figure 4.

### 2.2. Attitude and Heading Determination from Open-Loop Gyros

The roll, pitch and yaw rate (*p*, *q*, and *r* respectively) of the vehicle are measured using rate gyros with respect to its body axis system. The relationship between rate gyro output and time rate of the Euler angles can be described using the direction cosine matrices as follows:

(1) $$\left[\begin{array}{c} \dot{\phi }\\ \dot{\theta }\\ \dot{\psi } \end{array}\right]\;=\;\left[\begin{array}{ccc} 1 & \text{sin}\;\phi \;\text{tan}\;\theta & \text{cos}\;\phi \;\text{tan}\;\theta \\ 0 & \text{cos}\;\phi & -\text{sin}\;\phi \\ 0 & \frac{\text{sin}\;\phi }{\text{cos}\;\theta } & \frac{\text{cos}\;\phi }{\text{cos}\;\theta } \end{array}\right]\left[\begin{array}{c} p\\ q\\ r \end{array}\right]$$

where *ϕ* is roll angle, *θ* is pitch angle, and *ψ* is yaw angle.

As a physical instrument, the rate gyro device also carries some errors such as axis misalignment, fixed bias, drift bias, fixed scale factor errors, asymmetric scale factor errors, and so on. The bias drift is one of the most serious errors deteriorating the accuracy of an AHRS. The bias drift, which shows normally nonlinear characteristics, causes the integration result to drift-off from the true attitude as a function of time and rapidly renders any calculations useless.

**Figure 5.**
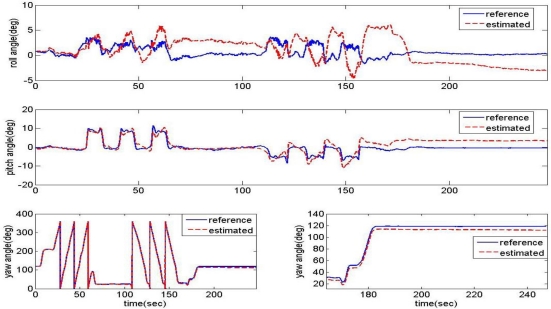
Attitude and heading determination from gyro measurement only.

Euler angles obtained from the open-loop integration process of Equation (1) using first order Euler method of integration are illustrated in Figure 5. This result shows that without correction the bias errors creating wandering attitude angles and the gradual instability of the integration drifting.

### 2.3. Attitude Determination from Open-Loop Accelerometers

Attitude angles *ϕ* and *θ* can also be obtained from a vehicle’s gravity vector. The following equation dictates a vehicle’s equation of motion in terms of specific forces (*f_x_*, *f_y_* and *f_z_*), which are the accelerometers reading in the body axes:

(2) $$\left[\begin{array}{c} f_{x}\\ f_{y}\\ f_{z} \end{array}\right]=\left[\begin{array}{c} \dot{u}\\ \dot{v}\\ \dot{w} \end{array}\right]+\left[\begin{array}{ccc} 0 & w & -v\\ -w & 0 & u\\ v & -u & 0 \end{array}\right]\left[\begin{array}{c} p\\ q\\ r \end{array}\right]+\left[\begin{array}{ccc} -r^{2}-q^{2} & \mathrm{pq}-r^{2} & \mathrm{pr}+\dot{q}\\ \mathrm{pq}+\dot{r} & -p^{2}-r^{2} & \mathrm{rq}-\dot{p}\\ \mathrm{pr}-\dot{q} & \mathrm{rq}+\dot{p} & -q^{2}-p^{2} \end{array}\right]\left[\begin{array}{l} l_{x}\\ l_{y}\\ l_{z} \end{array}\right]+g\left[\begin{array}{c} \text{sin}\;\theta \\ -\text{cos}\;\theta \text{sin}\;\phi \\ -\text{cos}\;\theta \text{cos}\;\phi \end{array}\right]$$

where, (*u*, *v*, *w*) and (*l_x_*, *l_y_*, *l_z_*) are linear velocity components and accelerometer’s coordinates along each axis in the body frame with its origin at the center of gravity, respectively. When all of the parameters of Equation (2), (*f_x_*, *f_y_*, *f_z_*, *u̇*, *v̇*, *w̄*, *u*, *v*, *w*, *p*, *q*, *r*), are measured in flight, near-true attitude angles can be determined. Normally, however, it is hard for a low-cost UAV to measure (*v*, *w*), and their time derivatives. Thus, with reduced sensor fit in the UAV, it is not easy to obtain true attitude measurements from accelerometers. Assuming that the UAV is driving steady-level states, linear acceleration terms integrate to zero over time and (*p*, *q*, *r*) can be neglected. With this simplification, Equation (2) may be simplified as follows:

(3) $$\phi =\;\text{atan}\;2(f_{y},f_{z})\;\;\;,\;\;\;\;\;\;\;\;\theta =\text{atan}\;2(-f_{x},\sqrt{f_{y}{}^{2}+f_{z}{}^{2}})$$

It should be noted that the attitude determination using Equation (3) is true only for specific dynamic conditions such as steady level flight. If an aircraft is circling for an extended period, the accelerometer will not only detect gravitational acceleration, but also centrifugal forces, resulting in an incorrect attitude determination. In addition, the change in transient forward acceleration is another possible error source. This means that the attitude calculation from accelerometers is not valid under all dynamic conditions.

**Figure 6.**
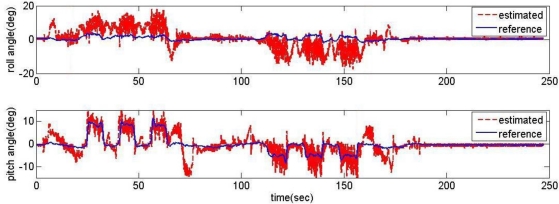
Attitude determination from accelerometer measurement only.

The attitude angles obtained from Equation (3) are illustrated in Figure 6 and compared with the reference from the vertical gyros. These results represent that the attitude from accelerometers tends to approximately follow the vertical gyro output in a steady level state, but shows large errors with noises and loose reliability under transient and high dynamic conditions.

### 2.4. Heading Determination from Open-Loop Magnetometers

A standard three-axis magnetometer reads the magnetic field in an aircraft’s body axis system as (*m_x_*, *m_y_* and *m_z_*). The magnetometer readings in relation to the Earth’s magnetic field vectors (*m_N_*, *m_E_* and *m_D_*) arranged in North, East and Down are as follows:

(4) $$\left[\begin{array}{c} m_{x}\\ m_{y}\\ m_{z} \end{array}\right]=\left[\begin{array}{ccc} \text{cos}\;\theta \text{cos}\;\psi & \text{cos}\;\theta \text{sin}\;\psi & -\text{sin}\;\psi \\ \text{sin}\;\phi \text{sin}\;\theta \text{cos}\;\psi -\text{cos}\;\phi \text{sin}\;\psi & \text{sin}\;\phi \text{sin}\;\theta \text{sin}\;\psi +\text{cos}\;\phi \text{cos}\;\psi & \text{sin}\;\phi \text{cos}\;\theta \\ \text{cos}\;\phi \text{sin}\;\theta \text{cos}\;\psi +\text{sin}\;\phi \text{sin}\;\psi & \text{cos}\;\phi \text{sin}\;\theta \text{sin}\;\psi -\text{sin}\;\phi \text{cos}\;\psi & \text{cos}\;\phi \text{cos}\;\theta \end{array}\right]\left[\begin{array}{l} m_{N}\\ m_{E}\\ m_{D} \end{array}\right]$$

Equation (4) shows how *ϕ*, *θ*, and *Ψ* are related to a magnetometer’s reading and geomagnetic field vectors toward North, East and Down. The yaw angle *ψ* refers to true magnetic north can be produced after going through a certain calculation provided the results of attitude estimations, *ϕ* and *θ*.

Resolving the geomagnetic vector onto a local tangent plane where the *x*-axis towards the North Pole will put the geomagnetic vector toward East is equal to zero. Hence, Equation (4) can be rearranged as:

(5) $$\left[\begin{array}{c} m_{N}\;\text{cos}\;\psi \\ m_{N}\;\text{sin}\;\psi \\ m_{D} \end{array}\right]=\;\left[\begin{array}{ccc} \text{cos}\;\theta & \text{sin}\;\theta \text{sin}\;\phi & \text{sin}\;\theta \text{cos}\;\phi \\ 0 & -\text{cos}\;\phi & \text{sin}\;\phi \\ -\text{sin}\;\theta & \text{cos}\;\theta \text{sin}\;\phi & \text{cos}\;\theta \text{cos}\;\phi \end{array}\right]\left[\begin{array}{c} m_{x}\\ m_{y}\\ m_{z} \end{array}\right]$$

hence, the division of first and second row gives:

(6) $$\psi =-\text{atan}\;2(Y_{h},X_{h})$$

where, *X_h_* = *m_N_* cos *ψ* and *Y_h_* = *m_N_* sin *ψ* represent the earth’s horizontal magnetic field components. Preserving the sign of the numerator and denominator of Equation (6) and using the appropriate logical expressions will give heading angle information for all conditions of attitude and magnetometer readings from 0 to 2π.

**Figure 7.**
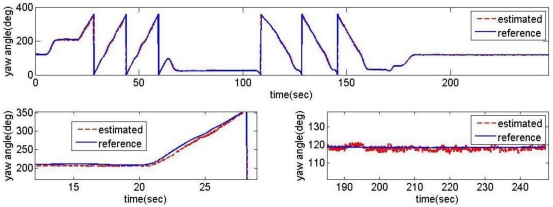
Heading angles calculated from magnetometer measurements.

The heading angles obtained from Equation (6) with the actual reading of magnetic vector (*m_x_*, *m_y_* and *m_z_*) and the vertical gyro information of *ϕ* and *θ* are illustrated in Figure 7. These results represent that the heading angle information from magnetometer tends to follow the reference output approximately in all test scenarios. However, the phase loss in high dynamic situations and the noisy conditions are shown in Figure 7 of the expanded scale.

## 3. Conventional Linear Complementary Filter

To observe and compensate for gyro drift, a process called augmentation, termed a complementary filter due to its frequency filtering properties, is used. The basic idea of the complementary filtering AHRS is: (1) to combine the outputs of gyro and accelerometer (for attitude estimate *ϕ* and *θ*), and (2) to combine the outputs of the gyro and magnetometer (for heading estimate *ψ*) to obtain a good estimate of the orientation, and thereby compensating for the drift of the rate gyro and for the slow dynamics of the accelerometer and magnetometer. To fuse the signal, we designed conventional complementary filters for the heading and attitude estimation, respectively. After having identified the dynamics of all sensors, we tried several complementary filters and could select the best one for heading estimation. However, it is shown that the limitation of the accelerometer (see Section 2.3) renders the conventional linear complementary filter alone unsuitable for the estimate of the attitude of the UAV.

### 3.1. Filtering Structure

**Figure 8.**
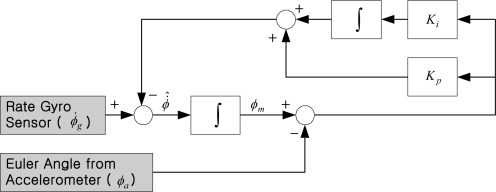
Conventional filtering estimator block diagram for roll axis channel.

Figure 8 shows the conventional filtering estimator block diagram for the roll axis channel. A similar block diagram could also apply to the pitch and heading channel. The role of the estimator is to compare attitude (or heading) angles resulting from the integration of the gyros with the attitude angle products from the accelerometers (or with heading angle products from magnetometer). The error between *ϕ_m_* and *ϕ_a_* is fed-back through a proportional and integral controller with a pair of estimator gains *K_p_* and *K_i_*.

The mathematical relation of the estimated attitude (*ϕ_m_*), the attitude products from the accelerometers (*ϕ_a_*), and the attitude rate products from the gyros (*ϕ̇_g_*) in the Laplace form (with Laplace operator *s*) are as follows:

(7) $$\phi_{m}=\frac{1}{s}{\dot{\phi }}_{g}+\frac{K_{p}}{s}\left(\phi_{a}-\phi_{m}\right)+\frac{K_{i}}{s^{2}}\left(\phi_{a}-\phi_{m}\right)$$

The error equation of the estimated attitude yields:

(8) $$\delta \phi_{m}=\frac{s\delta {\dot{\phi }}_{g}+(K_{p}s+K_{i})\delta \phi_{a}}{s^{2}+K_{p}s+K_{i}}$$

Applying the final value theorem to Equation (8), we can see that the estimated attitude error converges to the error of the angle calculated from accelerometer:

(9) $$\underset{t\to \infty }{\text{lim}}\delta \phi_{m}=\delta \phi_{a}$$

The controller gains, *K_p_* and *K_i_*, are chosen by relating them to the cut-off frequency (*ω*) and damping ratio (*ς*) of the estimator as:

(10) $$K_{i}=\omega^{2}\;\;,\;\;K_{p}=2\varsigma \omega$$

During the investigation, the damping ratio *ς* is fixed to a suitable value of 0.707 to provide a good transient response. Hence:

(11) $$K_{p}=\sqrt{2}\omega$$

Therefore, the system is only characterized by the cut-off frequency, and this should be chosen appropriately to optimize the process.

### 3.2. Design of the Complementary Filter for Heading Estimate

As noted in Section 2.4, the magnetometer shows phase loss under a high dynamic condition. Thus, the basic requirements for the filter are: (1) to exhibit constant amplification and small phase loss up to frequencies well above the cut-off frequency of the magnetometer and (2) to use magnetometer in the widest possible ranges of frequencies in order to keep the sensitivity to offset of the rate gyro at a minimum.

**Figure 9.**
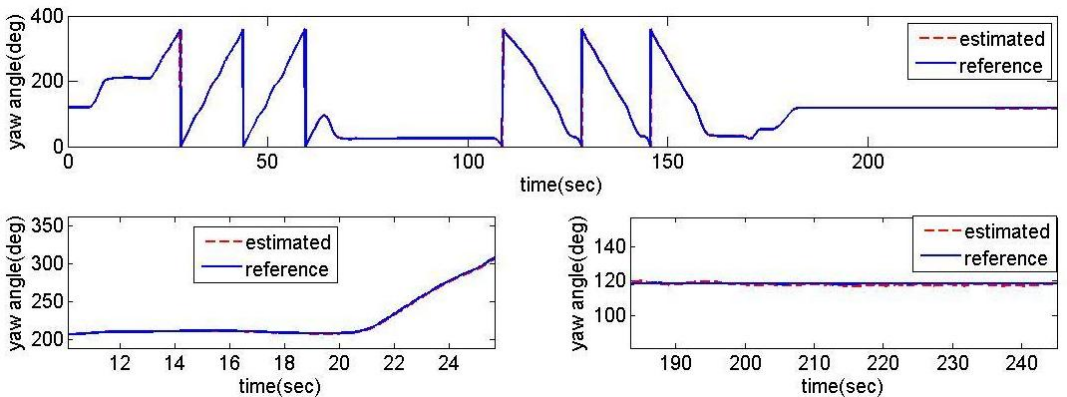
Heading angles from the filtering estimator.

To decouple the effectiveness of the heading filter with attitude ones, the attitude information (*ϕ* and *θ*) that is needed to go through a certain calculation of Equation (5) is provided by the reference vertical gyro. After having identified the dynamics of all sensors, we tried several complementary filters and could select the best one. The estimator’s cut-off frequency had been set at 0.1 rad/s. The heading angles obtained from the filtering estimator is illustrated in Figure 9. These results represent that the heading estimates tend to follow the reference output satisfactorily in all conditions, showing the noises and phase losses of magnetometer are minimized.

### 3.3. Problems of the Linear Complementary Filter for Attitude Estimate

**Figure 10.**
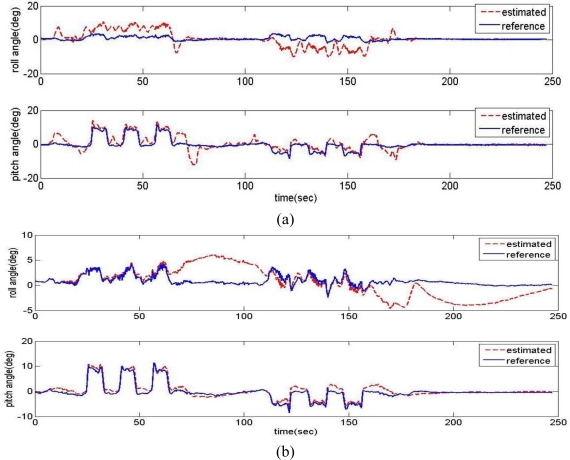
Effects of estimation filter cut-off frequency on estimated attitude angles. **(a)** When cut-off frequency = 1 rad/s. **(b)** when cut-off frequency = 0.05 rad/s.

Variations in the estimator’s cut-off frequency (natural frequency) had been tested at 1 rad/s and 0.05 rad/s. The estimation results are compared with the vertical gyro outputs in Figure 10. When the cut-off frequency is 1 rad/s (a relatively high frequency), the estimation angles converge well at the steady state, but not well during high dynamic motion. Though the cut-off frequency is set to a lower frequency, 0.05 rad/s, the estimation results are not improved on the whole.

As the cut-off frequency goes lower, the high frequency dynamic characteristic of the filter becomes better, but the low frequency one becomes worse. So, there should be an optimization problem of selecting a cut-off frequency value to improve the performance of the estimation filter over a wide range of dynamic conditions. However, it seems quite difficult to achieve acceptable performance under all dynamic conditions using this fixed gains linear filtering structure, especially with low-cost (high bias drift) MEMS sensors. To surmount these typical problems and drawbacks, it is necessary to expand the current attitude estimation algorithm so as to have an adaptive function under varying flight dynamics.

## 4. Gain-Scheduled Complementary Filter for Attitude Estimate

To overcome the typical problems and drawbacks of the fixed gain filtering structure for the attitude estimate presented in Section 3.3, a gain-scheduled complementary filter is considered. By using switching architecture inference, each parameter of the filtering estimator of the pitch and roll channels is determined adaptively under varying flight dynamics. For this solution, switching logics are based on system dynamics sensed by the accelerometers to yield optimal performance.

### 4.1. Tuning of Parameters Based on Switching Logic

**Figure 11.**
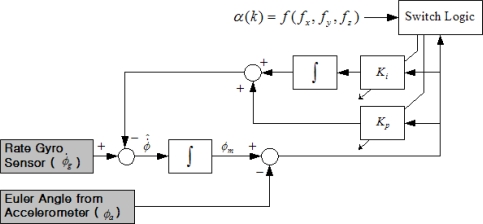
Gain-scheduled complementary filter for attitude estimate.

Figure 11 shows a block diagram for the gain-scheduled attitude filtering estimator augmented by switching logic for roll axis channel. The same block diagram could also apply to the pitch axis channel. The approach taken here is to exploit switching logic to generate proper parameters of the filtering estimator according to varying dynamic conditions. As seen earlier in Equations (10) and (11), the parameters are determined only by the cut-off frequency. Using simulation study results, the nominal cut-off frequencies are set at 0.05 rad/s and 0.01 rad/s for roll and pitch channel, respectively.

In the proposed scheme, the cut-off frequency is switched to the predetermined value according to the acceleration levels (non-acceleration, low-acceleration, and high-acceleration mode) determined by the scalar dynamic acceleration *α*(*k*), defined as:

(12) $$\alpha (k)=\left|\sqrt{f_{x}{\left(k\right)}^{2}+f_{y}{\left(k\right)}^{2}+f_{z}{\left(k\right)}^{2}}-g\right|$$

The threshold values of the scalar dynamic acceleration *α*(*k*) to identify the acceleration level are extracted experimentally based on the characteristics of gyros and accelerometers and the design requirements of the applications. The switching logic for the filter gain has the following scenarios:

- Non-acceleration mode: In this mode, *α*(*k*) < 0.015 g, the accelerometer measurement of the gravity has observability and yield good estimates of the attitude. To assign more weighting to accelerometers, the cut-off frequency is set at 0.1 rad/s for both roll and pitch axes.
- Low-acceleration mode: In this mode, 0.015 g < *α*(*k*) < 5 g, the uncertainty of the acceleration for the attitude estimation should be considered. The nominal cut-off frequencies are set at 0.05 rad/s and 0.01 rad/s for roll and pitch channel, respectively.
- High-acceleration mode: In this mode, 5 g > *α*(*k*), the system is in high dynamics, and the attitude estimation based on accelerometer measures of the gravity is far from accurate. Therefore, the cut-off frequency is set to 0 such that the accelerometer feedback was turned off; hence the weighting is fully imposed to the gyros.

### 4.2. Experimental Result

**Figure 12.**
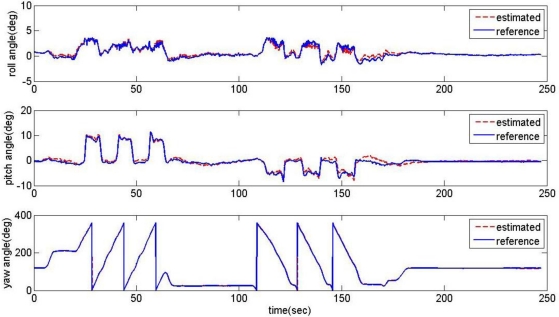
Response of the acceleration based switching architecture AHRS.

**Figure 13.**
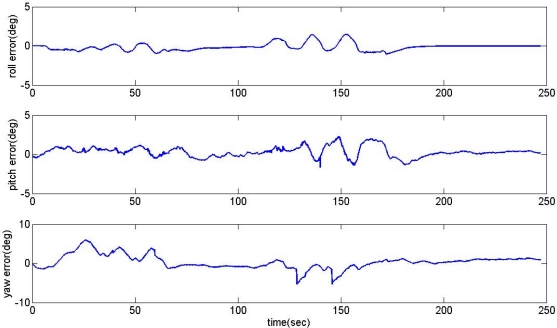
The error between reference and filtering.

The proposed acceleration based switching architecture has been tested in the same situations with conventional estimator in Section 2. The measurements of low-cost MEMS inertial and magnetic sensors output obtained during the experimental road captive test are used for the implementation of the estimation scheme. The time responses for roll, pitch, and heading estimates are plotted in Figure 12. The results obtained from the high-precision vertical gyro are also presented for comparison. The error between reference and filtering is provided in Figure 13.

**Figure 14.**
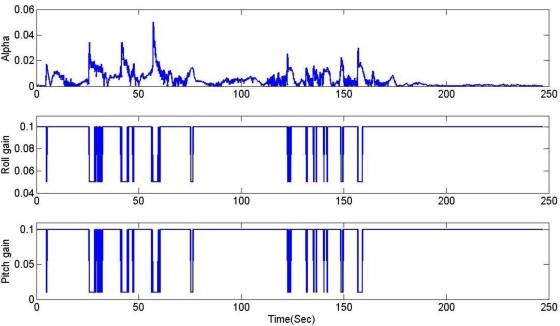
Gains switched online as a function of the scalar dynamic acceleration *α*(*k*).

It is obvious that these results are much better than the conventional filtering estimator results shown in Figure 9. In order to verify the effectiveness of the estimation, the levels of agreement between the ‘truth model’ and the scheme results are calculated. The calculated errors based upon a simple root mean square (RMS) method are 0.2214, 0.6720, 2.0788 deg for roll, pitch, and heading, respectively. These performances are very encouraging since the estimation process was performed with low-cost MEMS inertial and magnetic sensors with a simple gain-scheduled complementary filter. Figure 14 shows the gains switched online as a function of the scalar dynamic acceleration *α*(*k*).

## 5. Conclusions

This paper describes a robust and simple algorithm for an attitude and heading reference system (AHRS) based on low-cost MEMS inertial and magnetic sensors. The proposed AHRS scheme uses acceleration based switch rules and reasoning to determine the filtering estimator parameters by adjusting the cut-off frequency. Although a rule of thumb for choosing the ranges for *α*(*k*) identifying acceleration level is obtained experimentally, it is still possible to make further performance improvements by fine tuning the ranges as well as by adjusting cut-off frequencies for each level. The proposed scheme has shown a good damping characteristic for the drift errors from the gyro measurement as well as the noise corruption from the accelerometer measurement in all dynamic conditions. The scheme gave an accuracy of better than 0.2214 deg in roll, 0.6720 deg in pitch and 2.0788 deg in heading compared to the ‘truth model’ of the vertical gyro reading. This performance is very encouraging since this indicates that the high accuracy AHRS should be possible with low-cost MEMS inertial and magnetic sensors with innovative computational methods.

## Figures and Tables

**Table 1.** System Specifications (ADIS16405).

	
	**Gyro**	**Accelerometer**	**Magnetometer**
Range : X, Y, Z	± 350(deg/s)	± 18(g)	± 3.5(gauss)
Sensitivity	0.05(°/sec/LSB)	3.33(mg/LSB)	0.5(mgauss/LSB)
Bias Stability	35(deg/h)	-	-
Angle/Velocity Random Walk	$2.0(\text{deg}/s/\sqrt{\mathrm{hr}})$	$0.2(m/s/\sqrt{\mathrm{hr}})$	-
